# New Appliances and Things Medical

**Published:** 1895-07-06

**Authors:** 


### NEW APPLIANCES AND THINGS MEDICAL.

[We shall be glad to receive, at our Office, 428, Strand, London, W.C., from the manufacturers, specimens of all new preparations and appliances which may be brought out from time to time.]

### BINIODIDE ANTISEPTIC SOAP.

(E. Cook and Co., Bow, London, E.)

Although this soap has been for many years before the public, its place among the antiseptic soaps has not been fully recognised by the profession. Unlike the majority of its competitors, its antiseptic properties are due to the presence of a metallic salt, and that one which was formerly regarded as incompatible with soap solutions. This has been effected by making use of the well-known property of mercury salts to form double compounds with the alkaline salts of the halogens. Under the Thompson patent the biniodide of mercury has been found the most suitable salt to employ, and Messrs. E. Cook and Sons now place this biniodide soap on the market in three strengths, viz., 3 per cent., 1 per cent., and ½ per cent. of biniodide. Dr. Sims Woodhead made a series of comparative experiments with this and other antiseptic soaps, and found that the sample he examined had "much more germicidal properties than any other antiseptic soap" he was able to procure. Since this report other manufacturers, notably in the United States, have also been successful in incorporating mercury salts with soap without decomposition, and they must hence now be recognised as formidable rivals of the older medicated soaps in which carbolic acid and other organic substances are the effective antiseptics employed. Dr. Sims Woodhead and Dr. Archibald have also tested this biniodide soap against the carbolate of mercury soap, which they find is not such a powerful disinfectant as the biniodide soap, and that its germicidal action is considerably slower. The samples sent us for examination have been analysed by our chemist, who finds that they are well-made soaps containing iodide of mercury combined with excess of potassium iodide, and that when dissolved in warm water there is no separation of any insoluble mercury compound. It is therefore to be inferred that the antiseptic and disinfectant properties of the soap remain active in use, and that consequently the experiments of Dr. Sims Woodhead upon this soap truly represent its efficiency in practice. After decomposing the soap with acid a solution of the biniodide of mercury is obtained which, when treated with iron alum, and dilute acid, evolves the iodine combined with the potassium present, and at the same time causes a separation of the red iodide of mercury which can then be separated and weighed. The results of the analysis of samples of the three grades of soap are as follows:—

| | Potassium Iodide. | | Mercuric Iodide. | | Biniodide $KHg_2I_5 + 2H_2O$. |
|---|---|---|---|---|---|
| 3 per cent. soap | 2·25 | ... | 2·39 | ... | 2·92 |
| 1 per cent. soap | 0·94 | ... | 0·63 | ... | 0·77 |
| 0·5 per cent. soap | 0·45 | ... | 0·26 | ... | 0 31 |

It will thus be seen that the quantity of germicide present is approximately graded to the several kinds of soap manufactured, and that it may be recommended for use in obstinate cases of ringworm, eczema, and scabies, and also as a general disinfectant soap for clothes and articles used in infectious disease cases. Although it is distinctly pointed out that it is a medicinal and not a toilet soap, we note that the fancy boxes containing the 1 per cent. and ½ per cent. tablets are marked Cook's Biniodide Toilet Soap. Although cases of mercury poisoning are happily rare (and far less frequent than those from carbolic acid), we cannot help pointing out the desirability of using such mercury soaps only when specially required and under medical directions, since should they be generally used an element of danger, if not of poisoning at least of salivation, would be introduced into the nursery and on to the toilet table.

